# Signal Transduction in Leaf Senescence: Progress and Perspective

**DOI:** 10.3390/plants8100405

**Published:** 2019-10-10

**Authors:** Salman Ahmad, Yongfeng Guo

**Affiliations:** 1 Tobacco Research Institute, Chinese Academy of Agricultural Sciences, Qingdao 266101, China; safitfa@yahoo.com; 2 Plant Breeding & Genetics Division, Nuclear Institute for Food & Agriculture, Tarnab, Peshawar P.O. Box 446, Pakistan

**Keywords:** leaf senescence, age, signal transduction, receptor-like kinase, LRR-RLKs, MAP kinase cascade, transcription factor

## Abstract

Leaf senescence is a degenerative process that is genetically controlled and involves nutrient remobilization prior to the death of leaf tissues. Age is a key developmental determinant of the process along with other senescence inducing factors. At the cellular level, different hormones, signaling molecules, and transcription factors contribute to the regulation of senescence. This review summarizes the recent progress in understanding the complexity of the senescence process with primary focuses on perception and transduction of senescence signals as well as downstream regulatory events. Future directions in this field and potential applications of related techniques in crop improvement will be discussed.

## 1. Introduction

Leaf senescence is the cumulative response of multiple factors operating at the final stages of the plant life cycle. The onset of senescence is attributed to the loss of chlorophyll in the cell and subsequently extends towards the disintegration of macromolecules including proteins, carbohydrates, lipids, and nucleic acids [1,2,3]. At the same time, the degenerated molecules are recycled and reallocated into storage tissues and organs depending on the life cycle of the plants. Plants with short life cycles restore the translocated nutrients into their seeds and fruits while in perennial plants like trees, the stem and root serve as storage organs to be utilized later on for the emergence of new leaves and flowers in the next season [4,5,6]. The gradual degradation of chloroplasts and loss of photosynthetic activities leave the yellow footprint on the leaf surface from the tip to the base. This preferential break down of chlorophyll leads to the color change of monocarpic plants from green to golden yellow as the grain matures before harvesting. Furthermore, chlorophyll breakdown in combination with synthesis of new pigments like anthocyanin and phenols due to hydrolysis of macromolecules give a conspicuous range of colors in the autumn leaves of deciduous trees [7].

Leaf senescence can be viewed as a process of cell degenration and death, but deep down it is a systematic mechanism that operates under tight genetic control to ensure the survival of the plant and accumulation of nutrients for the next generation. The initiation of senescence, genetic diversity in symptoms of senescence, and translocation of the nutrients temporally, spatially, and quantitatively are the manifestations of a genetically controlled program. At the cellular and subcellular levels, different hormones, receptors, and transcription factors contribute to the regulation and execution of senescence. Genetic and molecular level studies translate a huge spectrum of the complex and highly organized senescence syndrome [8,9]. Genetic approaches focusing on mutants with altered leaf phenotypic expression during senescence suggested a significant role of regulatory elements, either positive or negative, for switching on and off the progression of senescence [10,11]. Exploring and characterization of inter and intracellular signaling pathways, regulatory factors, and differential gene expression profiles give a comprehensive molecular insight of the senescence syndrome. Signal transduction is a very important aspect of leaf senescence as the initiation or induction of the process is directly influenced by different environmental or internal cues/signals. Drought, salinity, high/low temperature, nutrient deficiency, low light intensity, and pathogen infection are different types of environmental cues that push the plant towards senescence. Besides external factors, endogenous hormones also contribute in the signaling cascades of senescence. The triggered pathways as a result of external and internal signaling factors formulate a complex network of leaf senescence regulation [8,9].

Here in this review, we summarize all those essential factors and components that are involved in signal transductions with a view of contemporary research and advancement made in this important developmental phase of senescence.

## 2. Hormones as Senescence-Regulating Signals

Once the cell perceives the cues/signals of senescence, a series of signal transduction events are triggered involving various signaling components at different levels of regulation, and significant interactions take place among the different signaling components [8,9]. Hormones have a significant role in the regulation of both age-dependent and stress-induced leaf senescence. Almost all of the major phytohormones have been reported to function in the senescence signaling pathways. Ethylene, abscisic acid, brassinosteroids, jasmonic acid, salicylic acid, cytokinins, auxin, and gibberellic acid have been evidently found to control leaf senescence in various studies [12,13,14].

Ethylene has multiple roles in different developmental processes such as cell proliferation, elongation, cell size, abscission and fruit ripening, senescence, and stress responses to abiotic/biotic factors [12]. Ethylene has a role in senescence-related signal transduction and has been known as a senescence promoting hormone [15]. The ethylene signaling cascade has been predicted to form a straight pathway, including membrane-bound receptors at one end followed by a number of positive/negative regulators and downstream transcription factors at the other end of the pathway [16]. Application of ethylene accelerates senescence in leaves and flowers while inhibiting the synthesis of ethylene results for a delay in senescence [17]. Gene expression studies revealed an increased abundance of transcripts related to ethylene synthesis and signaling during age-dependent leaf senescence [16]. The mutant *ethylene-insensitive2* (*ein2*) plants exhibited delayed developmental leaf senescence due to low expression of 21 senescence-associated genes (*SAGs*) that encode the polygalacturonases and pectinesterases enzymes responsible for cell wall decomposition [18]. EIN3, a signaling molecule downstream from EIN2, has been shown to positively regulate leaf senescence via activating two senescence-promoting transcription factors, ORE1 and AtNAP [19]. It has been confirmed that the ethylene signaling cascade mediated senescence functions in an age-dependent manner, evident by the treatment of exogenous ethylene on mature and young leaves, which resulted in the early and normal senescence in the respective leaves [12,20].

Abscisic acid (ABA) is an essential phytohormone regulating a wide range of growth and development processes including germination, dormancy, maturity, embryogenesis, stomatal closure, shoot and root growth regulation, fruit ripening, leaf senescence, abscission, and stress responses that include drought, salinity, and low temperature [12,21]. The mechanisms of these functions are controlled by different signal gears, i.e., Ca^2+^, ROS, G-Protein, SnRK2, PP2C, and MAPK pathways [14,22,23]. A number of studies on ABA clearly demonstrate its role as a positive regulator of leaf senescence. Microarray studies have revealed increased expression of genes involved in biosynthesis and signaling responses of ABA during senescence in Arabidopsis [18]. Studies also reported the increased level of endogenous ABA and the upregulation of genes associated with ABA signaling during senescence in different plants such as maize, tobacco, rice, oat, and Arabidopsis [11,24]. ABA receptor PYL9 has been shown to promote both drought resistance and leaf senescence in Arabidopsis [25]. Under dehydration conditions, ABA controls water loss via stomatal closure and prevents the plant from early senescence [26]. Senescence-associated gene 113 (*SAG113*) that encodes a protein phosphatase 2C (PP2C) acts as a negative regulator of ABA signaling by suppressing stomatal closure, which results in the desiccation of the leaves [12,24,27]. 

Brassinosteroids (BR) have multifunctional roles in plants such as germination, flowering, abscission, senescence, and in response to various stresses [12,28,29]. The BR signaling pathway consists of three BRI1-associated receptor kinase 1 (BAK1) homologs acting as co-receptors of the membrane-bound receptor kinase 1 (BRI1) to perceive BR ligands [28]. The role of brassinosteroids in the positive regulation of leaf senescence has been marked by the acceleration of senescence through external treatment and delayed senescence in BR mutant plants [12,30]. The application of epibrassinolide (eBL) on detached leaves of wheat exhibited delayed and accelerated leaf senescence provided in low and high concentration, respectively [30]. The expression of genes encoding BR signaling proteins BRI1 kinase inhibitor 1 (BKI1) and BZR1 in senescing leaves was found to be significantly suppressed in the rice early senescence mutant *ospls1* [31]. When detached leaves of *Pisum sativum* were treated with 24-epibrassinolide (EBR), senescence was promoted and free fatty acid contents were increased, suggesting that BRs might regulate leaf senescence via altering cell lipid composition [32].

Jasmonic acid (JA) and methyl jasmonates (MeJA) are involved in the different processes of plant growth and development including senescence and various biotic and abiotic stress responses [12,33]. The involvement of jasmonate in senescence regulation was first observed when MeJA treated detached oat leaves exhibited accelerated leaf senescence [34]. In Arabidopsis 14 of the 125, senescence-associated enhancer trap lines showed JA-induced reporter gene expression [35]. Four times more endogenous JA was produced in senescing leaves compared to non-senescing ones, and JA treatments initiated senescence in attached and detached leaves [36]. Increased expression of JA synthesis genes (*LOX3*, *OPR3*, *AOC1*, and *AOC4*) and signaling genes (*MYC2*, *JAZ1*, *JAZ6*, and *JAZ8*) were reported during the age-dependent senescence of leaves [16,37]. Reduced expression of photosynthesis-associated genes occurred after exogenous application of JA and MeJA [12]. MeJA application also increased the expression of genes associated with age-dependent senescence including *SEN4*, *ERD1*, and *SAG21* [12,38,39]. An ethylene response factor, BrERF72, has been suggested to be involved in the regulation of JA-mediated activation for *BrLOX4*, *BrAOC3*, and *BrOPR3* expression during JA-promoted leaf senescence in Chinese flowering cabbage [40].

Salicylic acid (SA) acts as a signaling molecule for regulating developmental processes including seed germination, flowering, senescence, and responses to abiotic/biotic stresses [41]. As a positive regulator of developmental leaf senescence, SA has been suggested to be involved in both the initiation and progression of leaf senescence [16,42]. Transcriptome and microarray analysis reported a number of SA biosynthesis, signaling, and responsive genes being upregulated during developmental leaf senescence [16]. A gene expression profiling study of the Arabidopsis wild type and mutant-NahG (with reduced SA) implied the dependence of many *SAGs* on the SA signaling pathways [16,18]. A more recent study suggested that the transcription factor WRKY75 positively regulates leaf senescence by promoting SA production and repressing H_2_O_2_ scavenging, partially by suppressing the transcription of *CATALASE2* [43]. 

Cytokinins (CKs) are important phytohormones with critical roles in the growth and developmental process of plants. CKs are also known as senescence retarding hormones, inhibiting chloroplast degradation when applied exogenously or endogenously [11,44]. The transcriptome analysis revealed that during senescence, decreased accumulation in the transcripts of the CK synthesis genes (*isopentyl phosphotransferase* (*IPT*) and *cytokinin synthase*) and increased levels of genes encoding the cytokinin deterioration enzymes, such as cytokinin oxidase and cytokinin inactivating N- and O-Glycosylases, were present [18]. When the CK synthesis gene *IPT* is expressed under the control of the senescence-specific SAG12 promoter, significant delay of leaf senescence has been observed in a large number of plant species [44,45]. Due to the fine-tuned senescence-specific expression of *IPT* in these studies, senescence-induced increase in endogenous CK levels suppressed or reversed leaf senescence once initiated [45]. In addition to natural senescence, elevated CK levels in the *SAG12-IPT* expressing plants often caused increased tolerance to stresses and delayed senescence induced by stress conditions [45]. Suppression of drought-induced leaf senescence was observed in creeping bentgrass with increased expression of *IPT*, which caused the activation of antioxidant enzymes including peroxidase, superoxide dismutase, and catalase [12,46]. Maintenance of photosynthetic activities and extension in root growth under moisture stresses has been observed in plants overexpressing cytokinin synthesis genes [46,47]. In Arabidopsis, the CK signaling pathway has been shown to include His protein kinases (AHKs) as the CK receptor, B-type phosphorelay carrier, and A-type nuclear response regulator (ARRs), which function downstream from the receptors by controlling leaf senescence [16,48]. A gain-of-function mutation in CK receptor AHK3 caused delayed leaf senescence, while a loss-of-function mutation in this protein conferred a reduced sensitivity to a cytokinin-dependent delay of leaf senescence [49]. In addition, 71 upregulated and 11 downregulated immediate CK responsive genes in Arabidopsis reported in a genome-wide expression profiling could be potentially involved in CK-mediated senescence signaling [50]. 

Auxins have a significant role in the growth and development of plants and have been suggested to act as a negative regulator of leaf senescence [51]. Treatment of detached leaves of Arabidopsis with auxin caused reduced expression of *SAG12*, the marker gene for developmental senescence [52]. Increased accumulation of endogenous auxin in Arabidopsis plants overexpressing the auxin biosynthetic gene *YUCCA6* showed a delay of leaf senescence and decreased expression of *SAGs* [53]. Another study revealed that when the small auxin-up RNA gene *SAUR39* was overexpressed in rice, reduced auxin transport and availability of free IAA were observed in transgenic plants that showed early senescence phenotypes [54]. Furthermore, repressors of auxin response genes, AUXIN RESPONSE FACTORs 1 and 2, have been identified to be involved in the regulation of senescence and the abscission of Arabidopsis leaves [55]. The Arabidopsis knockout mutant of *ORESARA14*, which encodes ARF2, displayed a delay in leaf senescence [56]. The role of auxin in senescence, however, appears to be more complex since increased level of auxin (IAA) in the senescence leaves has been reported [57], and IAA induced ethylene production in tobacco leaves was reported to be able to confront the senescence delaying activity of IAA [58].

Gibberellic acid (GA) promotes stem and leaf elongation, seed germination, flowering, fruit and seed formation, and responses to different stresses [59,60]. GA has been reported as a senescence retarding phytohormone, as the level of its active form decreases during the progression of developmental senescence [13]. The role of GA in delaying senescence was first shown by treating detached leaf tissues of *Taraxacum officinale* with GA [61]. A number of studies indicate the availability of free GA (GA4 and GA7) inhibited leaf senescence; although, one study suggested that the delayed leaf senescence in *Paris polyphylla* by GA was due to the provoking effects of ABA [62,63]. The expression of GA-inducible AtGA2OX2, which encodes GA 2-oxidase2 that deactivates GA, was reported to be increased 18-fold during senescence [16]. Arabidopsis mutants deficient in GA biosynthesis or GA signaling displayed delayed senescence phenotypes, supporting the role of GA as a negative player in regulating leaf senescence.

## 3. Transcription Factors in the Senescence Signal Transduction Pathway

Perception and transduction of the senescence-inducing cues/signals lead to drastic changes in gene expression, which drive the execution of the senescence syndrome and eventually lead to cell death [37,64,65]. Genes that are upregulated during senescence are generally termed as senescence-associated genes (*SAGs*), while genes with downregulated expression patterns, such as photosynthetic genes, are termed as senescence downregulated genes (*SDGs*). Transcription factors (TFs), as regulatory proteins serving as switches in the process of differential gene expression, have been shown to be involved in both the promotion and/or inhibition of plant senescence [9,66,67]. A number of TF families have been reported to be regulators of leaf senescence [18,27,68]. Based on DNA-binding domains, among the 2403 TF-encoding genes on the Arabidopsis genome, at least 287 TFs in 34 gene families have been shown to be senescence-associated, including members in the NAC, WRKY, MYB, C2H2, APE2, bZIP, and HB gene families [27,68,69,70]. 

NAC is a large TF family and has been widely associated with the regulation of the leaf senescence process [19,71,72,73]. Some NAC TFs genes like AtNAP, ORE1, ORS1, ANAC016, and ATAF1 are positive regulators of senescence, and their overexpression usually results in precocious senescence, while suppression of their expression exhibits delayed senescence in Arabidopsis [64,74,75,76]. Contrarily, the negative regulators of senescence in the NAC family that give delayed senescence phenotype when overexpressed have also been reported, such as VNI2 (VND- INTERACTING2) and JUB1 (JUNGBRUNNEN1) [77,78,79]. During senescence, NAC TFs usually form complex regulatory networks by controlling other NACs and interacting with NACs or other TFs to regulate the expression of the target genes [73,80].

Another important TF family that regulates leaf senescence is WRKY, which is involved in multiple growth and developmental processes, including senescence and stress responses. The roles of WRKY6, WRKY18, and WRKY22/WRKY29 have been reported in defense responses and senescence regulation [68,81,82,83]. WRKY53 is a senescence promoting transcription factor that directs the expression of a number of *SAGs*, some of which are related to stress responses and pathogen attacks [84,85]. WRKY22 has been identified as a target of the WRKY53 transcription factor, which has a positive role in the regulation of dark-induced senescence, while its knockout mutant exhibited delayed leaf senescence [85,86]. In addition, WRKY54 and WRKY70 have been confirmed to have a negative role in the regulation of senescence [42,85].

Members from other transcription factor families that regulate senescence include positive regulators AP2, AUXIN RESPONSE FACTOR ARF2, C-repeat binding factor CBF2, G-Box binding factor GBF1, MYB-like transcription factor AtMYBL [56,64,87,88], and negative regulators such as the B-type cytokinin response regulator ARR2 [49,64].

## 4. Perception and Transduction of Senescence-Related Signals

During the last two decades, the process of leaf senescence has been extensively studied in the model plant Arabidopsis with much emphasis on the role of different hormones and the identification of regulatory genes, especially those encode transcription factors [8,9]. However, the molecular mechanisms of how senescence cues/signals are sensed/perceived and transduced into the cell and ultimately lead to the switch of gene expression changes and drive the execution of leaf senescence are still unclear. In addition to age, which is believed to be the primary cue in plant senescence, a number of environmental stimuli also play a role in the triggering of senescence. These include drought, nutrient deficiency, darkness, extreme temperatures, pathogen infection, and plant hormones including ethylene, abscisic acid, jasmonic acid, and salicylic acid [44,89,90]. Perception and transduction of different cues/signals eventually leads to the similar “senescence sydrome” [64], and significant overlap and cross-talks between these signalling pathways are expected.

Many of these cues/signals are sensed/perceived by receptor proteins on the surface of the cell and then transduced across the plasma membrane to activate signal transduction inside the cell to activate the gene regulatory network of senescence. Information about the age and environment-mediated signal pathways in senescence is limited and unable to uncover the complex regulatory network of the process. However a large number of genes potentially involved in signal trasduction have been identified via transcriptomic analyses. The major groups of these genes include mitogen-activated protein kinase (MAPK) and receptor-like kinases (RLKs) [37,65,68].

### 4.1. Receptor-Like Kinases (RLKs) in Leaf Senescence

Plant receptor-like kinases (RLKs) are cell-surface receptors that have unique structural features [91,92]. A RLK generally has an N-terminal extracellular binding domain for ligand binding, a transmembrane domain that spans the plasma membrane, and a cytoplasmic kinase domain that usually functions in signal transduction via the phosphorylation of downstream components to activate the regulatory network [91]. The extracellular domain is involved in the perception of ligands [68,93]. Subsequently, after binding of the ligands, the functional feature of RLKs is to autophosphorylate the intracellular component that activates the downstream regulatory network and gene expression changes [82,93,94]. With more than 600 members encoded by the Arabidopsis genome, RLKs represent the largest superfamily of proteins in plants with widespread functional roles in development, growth, resistance to pathogens, self-incompatibility, and hormone responses [95]. More than 20 different types of extracellular domains of RLKs have been characterized, including leucine-rich repeats (LRR), epidermal growth factor repeats, self-incompatibility (S), and lectin domains [93,96,97]. The largest subfamily of RLKs in Arabidopsis is leucine-rich repeat receptor-like protein kinase (LRR-RLK) that has more than 200 members [95]. The extracellular binding domain of LRR-RLKs contains various numbers of leucine-rich repeat units, which are usually 24 amino acid-long [91]. Although a large number of LRR-RLK proteins have been identified in various plant species including Arabidopsis, tomato, rice, potato, and poplar [95,98,99,100], up to now, only a limited number of LRR-RLKs have been characterized to play a role in plant development and stress responses [91].

RLKs function by perceiving signals at the cell surface and transducing the signals across the plasma membrane to activate signal transduction inside the cell, which makes them good candidates of receptors for senescence-inducing signals. In fact, a number of proteins in the LRR-RLK family have been characterized as regulator of leaf senescence. Using the mRNA differential display approach, an LRR-RLK gene named senescence-associated receptor-like kinase (SARK) was isolated in bean (*Phaseolus vulgaris* cv Bulgarian) in order to have increased levels of both transcript and protein accumulation during leaf senescence [101]. The *P_SARK_* promoter has been used to drive *IPT* expression and to promote stress tolerance in plants [102]. The function of SARK in senescence regulation, however, was not reported. Another senescence-upregulated LRR-RLK gene named GmSARK was later isolated in the soybean (*Glycine max*) and was found to be involved in regulating leaf senescence [103]. The soybean GmSARK and its homolog in Arabidopsis, AtSARK, have both been shown to fuction as a positive regulator of leaf senescence. Inducible overexpression of *GmSARK* or *AtSARK* caused precocious senescence, whereas leaf senescence was delayed in plants with reduced expression of these genes [85,104]. More recently, an LRR-RLK protein from moss (*Physcomitrella patens*) homologous to the bean SARK, named PpSARK, was shown to function as a negative regulator of moss senescence [105]. It should be noted that although all the above-mentioned *SARKs* were reported to be senescence-upregulated, only PpSARK was isolated based on sequence similarity, and it shares relatively high homology with the bean SARK [104,105].

In addition to the SARKs, a number of other LRR-RLK proteins have been characterized for their roles in leaf senescence in Arabidopsis. An ABA-perceiving membrane-bound receptor kinase RPK1 (RECEPTOR PROTEIN KINASE 1) was identified to have a positive regulatory function in age-dependent and ABA-mediated leaf senescence [85,106]. Knock-out mutants of RPK1 displayed significant delay in both age-dependent and ABA-induced senescence [106]. More recently, a member from somatic embryogenesis receptor-like kinase subfamily LRR-RLK, SERK4, was found to act as a negative regulator in the leaf senescence signaling pathways [107]. The Arabidopsis SERK family consists of five members (SERK1-5), and the SERK proteins have been reported to function as co-receptors in various signaling pathways, regulating different processes in development and stress responses [108]. Interestingly, AtSARK was also identified as CLAVATA3 INSENSITIVE RECEPTOR KINASE 3 (CIK3), together with LRR II-RLKs CIK1, CIK2, and CIK4 in the same subgroup, to function as co-receptors in the CLAVATA pathway for regulating stem cell homeostasis in Arabidopsis [109]. It has been shown that one co-receptor can function with multiple receptors in different signaling pathways [108,110]. AtSARK and SERK4 are likely part of receptor complexes functioning together with other LRR-RLKs in regulating senescence. 

LRR-RLK senescence induced receptor-like kinase (SIRK) was characterized to show a leaf senescence-specific expression pattern [82]. More than 40 receptor-like kinase genes were identified to be associated with Arabidopsis leaf senescence [68]. A recent study identified a rice receptor kinase OSBBS1/OsRLCK109, which belongs to the RLCK subfamily and lacks an extra cellular domain, that plays important regulatory roles in leaf senescence and salt stress responses. The *bbs1* mutant exhibited hypersensitivity to salt stress and early leaf senescence phenotypes [111]. The roles of more LRR-RLKs and other types of RLKs remain to be elucidated.

### 4.2. Mitogen-Activated Protein Kinase in Senescence Signaling

Signal transduction initiated by RLKs is often carried out inside the cell via phosphorelay. The mitogen-activated protein kinase (MAPK) cascade (MAPKKK-MAPKK-MAPK) is one of the most important signal transduction pathways in plants. The involvement of the MAPK cascade pathways in a wide range of cellular processes, like cell division, differentiation, responses to biotic/abiotic stresses, and hormones, has been shown previously [112]. An earlier study identified 9 MAPKKK, 3 MAPKK, and 3 MAPK genes as being associated with Arabidopsis leaf senescence [68]. In maize, *ZmMPK5* was identified to be associated with leaf senescence and in recovery from low-temperature stress [113].

In Arabidopsis, MAPKKK18 was reported to be a positive regulator of leaf senescence, and the senescence regulatory role of MAPKKK18 was dependent on its kinase activity and ABA signaling [14]. Similarly, in rice, SPOTTED LEAF3 (SPL3), also kown as OsMAPKKK1, positively regulated leaf senescence via the ABA signaling pathway [114]. For MAPKK, MAPKK9 (MKK9) has been shown to play an important regulatory role in Arabidopsis leaf senescence. When *MKK9*, but not the kinase inactive form *MKK9KR*, was overexpressed, the transgenic plants displayed a premature leaf senescence phenotype. On the other hand, senescence in detached leaves of *mkk9* null mutant plants was delayed [90]. Another MAPKK in Arabidopsis, EDR1, was identified as a negative regulator of defense responses and ethylene-induced leaf senescence [115,116]. A maize MAPKK, ZmMEK1, was also characterized as a negative regulator of senescence. Accumulation of *ZmMEK1* transcripts was induced during dark-induced maize leaf senescence, and the expression of a dominant negative mutant of ZmMEK1 in Arabidopsis induced SA-dependent leaf senescence [117]. Signals are usually further transduced to MAPKs through phosphorylation before reaching transcription factors, which function to change gene expression. In Arabidopsis, MAPK6 (MPK6) was identified as the target of MKK9 in the MAPK cascade. When both *MKK9* and *MPK6* were expressed in protoplasts of the *mkk9* null mutant, MKK9 was shown to be able to phosphorylate MPK6 in Arabidopsis. The kinase activity was enhanced by the presence of a constitutively active form of MPKK9 (MKK9EE). And more importantly, *mpk6* null mutants phenocopied *mkk9* with delayed leaf senescence, and the senescence-promoting role of MKK9 was partially dependent on MPK6 [90]. In maize, the MAPK ZmSIMK1 was identified as the direct target of MAPKK ZmMEK1, and the ZmMEK1-ZmSIMK1 cascade and its modulating of SA levels were shown to play important roles in regulating leaf senescence [117]. 

The MAP kinase signalling cascade enentually changes gene expression through the function of transcription factors. Arabidopsis MPK6 was shown to function by promoting cleavage and nuclear translocation of ORESARA3 (ORE3)/ETHYLENE INSENSITIVE2 (EIN2), a protein involved in leaf senescence mediated by multople factors including ABA, ethylene, MeJA, age, and darkness. The released C-terminal end of ORE3/EIN2 (CEND) stabilized EIN3, a transcription factor that accelerates MeJA-induced leaf senescence [118]. Interestingly Arabidopsis MAPKKK MEKK1 was identified as a DNA-binding protein that directly regulates the senescence-promoting transcription factor WRKY53 [119]. 

### 4.3. Other Components in Senescence Signaling

In addition to the MAP kinase cascade, other signling systems such as the Calcium (Ca2+)-related signaling also participate in regulating leaf senescence. The Arabidopsis Bax inhibitor-1 (AtBI1) was shown to function in delaying MeJA-induced leaf senescence by suppressing the [Ca^2+^] cyt-dependent activation of MPK6 [120]. The Calcineurin B-like-interacting protein kinase 14 (CIPK14) interacted with and phosphorylated the ssDNA binding protein WHIRLY1 in Arabidopsis [121]. WHIRLY1 has been identified as a plastid-nucleus-located protein, which plays a role in regulating leaf senescence [122]. Phosphorylation of WHIRLY1 by CIPK14 resulted in increased accumulation of the protein in the nucleus and enhanced binding with the promoter of the senescence regulating transcription factor WRKY53 [121]. 

A phosphatase 2C-type protein phosphatase, SENESCENCE-SUPPRESSED PROTEIN PHOSPHATASE (SSPP), was identified as a signaling component functioning downstream from the LRR-RLK AtSARK. SSPP was found to be able to interact with and dephosphorylate the cytoplasmic domain of AtSARK. Overexpression of *SSPP* could rescue the precocious leaf senescence and changes in hormonal responses induced by AtSARK [123]. Another protein phosphatase 2C, SAG113, was identified as a direct target gene of the AtNAP transcription factor. SAG113 was characterized as a negative regulator of ABA signaling, which is specifically involved in controlling water loss during the process of leaf senescence [24,27].

## 5. Conclusions and Perspectives

**Figure 1:**
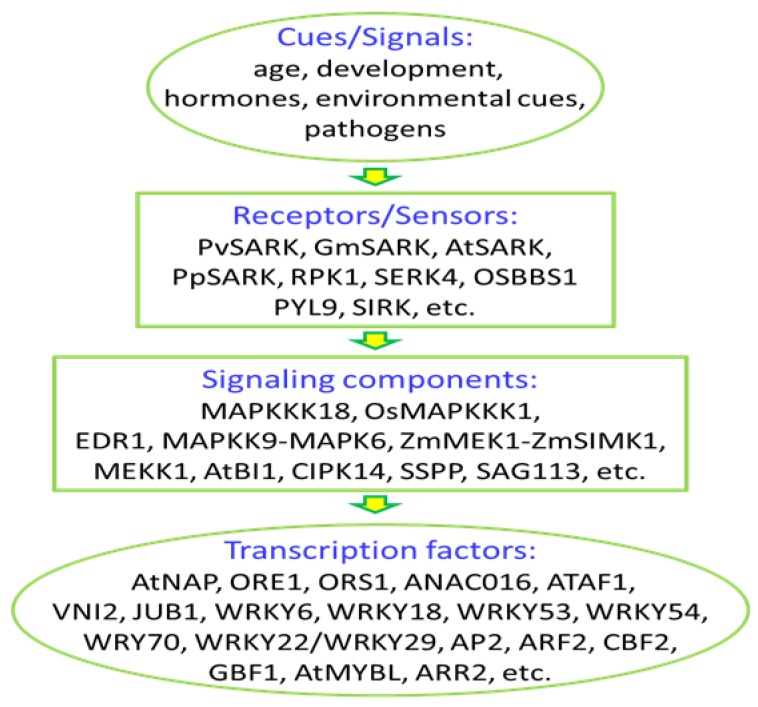
Signaling components identified in the process of leaf senescence.

Signal transduction is the critical phase of leaf senescence program, determining the activation of the downstream regulatory network of senescence. In the past two decades, the understanding of leaf senescence regulation has achieved a great deal, especially in hormonal regulation and transcriptional regulation of leaf senescence. A lot of work in signal perception and transduction was done, and large numbers of signalling components have been identified (Figure 1). However, only a very limited number of signalling cascades, such as the AtSARK-SSPP cascade [123], the MKK9-MPK6 cascade [90] in Arabidopsis, and the ZmMEK1-ZmSIMK1 cascade in maize [117], have been characterized. Much work is needed to elucidate the complexity and details of the leaf senescence regulatory network in-between signals and transcription factors by identifying more signaling proteins and perhaps more importantly, by buiding connections between different components that have been already identified. Some of the critical questions in signal transduction of leaf senescence remain unsolved. For example, what is the signal of aging, how the cue/signal of aging is perceived/sensed by the plant cells, and how this is transduced to transcription factors to change gene expression.

The available identified signal transducing components may provide a useful platform to formulate various strategies or systems to manipulate leaf senescence for agriculture production. Based on the well-studied homonal control and transcriptional control of leaf senescence, senecence-manipulating technologies have been developed for delaying leaf senescence, increasing stress torelance, and increasing crop yield. The *SAG12-IPT* system has been used to effectively delay leaf senescence in more than 20 plant species, and the NAP transcription factor-based technology has also been applied in more than a dozen plant species [45]. A number of the senescence-regulating proteins in the MAP kinase cascade have been identified as also being involved in stress responses [14,90]. Genetic manipulation of the signaling components at the junctions of cross talks between senescence and stress responses could potentially lead to increased stress tolerance and delayed senescence at the same time. On the other hand, with more and more signaling components of the leaf senescence regulatory network being identified and more complete signaling pathways unraveled, senescence manipulationg strategies with more specificity and even higher efficiency are expected to be developed for agricultual improvement. 

## References

[1] Soltabayeva A., Srivastava S., Kurmanbayeva A., Bekturova A., Fluhr R., Sagi M. (2018). Early senescence in older leaves of low nitrate-grown Atxdh1 uncovers a role for purine catabolism in N supply. Plant Physiol..

[2] Buet A., Costa M.L., Martínez D.E., Guiamet J.J. (2019). Chloroplast protein degradation in senescing leaves: Proteases and lytic compartments. Front. Plant Sci..

[3] Tamary E., Nevo R., Naveh L., Levin-Zaidman S., Kiss V., Savidor A., Levin Y., Eyal Y., Reich Z., Adam Z. (2019). Chlorophyll catabolism precedes changes in chloroplast structure and proteome during leaf senescence. Plant Direct.

[4] Maillard A., Diquélou S., Billard V., Laîné P., Garnica M., Prudent M., Garcia-Mina J.-M., Yvin J.-C., Ourry A. (2015). Leaf mineral nutrient remobilization during leaf senescence and modulation by nutrient deficiency. Front. Plant Sci..

[5] Havé M., Marmagne A., Chardon F., Masclaux-Daubresse C. (2016). Nitrogen remobilization during leaf senescence: Lessons from *Arabidopsis* to crops. J. Exp. Bot..

[6] Gortari F., Guiamet J.J., Cortizo S.C., Graciano C. (2018). Poplar leaf rust reduces dry mass accumulation and internal nitrogen recycling more markedly under low soil nitrogen availability, and decreases growth in the following spring. Tree Physiol..

[7] Kuai B., Chen J., Hörtensteiner S. (2017). The biochemistry and molecular biology of chlorophyll breakdown. J. Exp. Bot..

[8] Woo H.R., Kim H.J., Lim P.O., Nam H.G. (2019). Leaf senescence: Ssystems and synamics aspects. Annu. Rev. Plant Biol..

[9] Ali A., Gao X., Guo Y. (2018). Initiation, progression, and genetic manipulation of leaf senescence. Methods Mol. Biol..

[10] Hensel L.L., Grbic V., Baumgarten D.A., Bleecker A.B. (1993). Developmental and age-related processes that influence the longevity and senescence of photosynthetic tissues in *Arabidopsis*. Plant Cell.

[11] Lim P.O., Kim H.J., Nam H.G. (2007). Leaf Senescence. Annu. Rev. Plant Biol..

[12] Jibran R., Hunter D., Dijkwel P. (2013). Hormonal regulation of leaf senescence through integration of developmental and stress signals. Plant Mol. Biol..

[13] Schippers J.H.M., Jing H.C., Hille J., Dijkwel P.P. (2007). Developmental and hormonal control of leaf senescence. Senescence Process. Plants.

[14] Matsuoka D., Yasufuku T., Furuya T., Nanmori T. (2015). An abscisic acid inducible *Arabidopsis* MAPKKK, MAPKKK18 regulates leaf senescence via its kinase activity. Plant Mol. Biol..

[15] Kim J., Chang C., Tucker M.L. (2015). To grow old: Regulatory role of ethylene and jasmonic acid in senescence. Front. Plant Sci..

[16] Van der Graaff E., Schwacke R., Schneider A., Desimone M., Flugge U.-I., Kunze R. (2006). Transcription analysis of *Arabidopsis* membrane transporters and hormone pathways during developmental and induced leaf senescence. Plant Physiol..

[17] Jing H.C., Schippers J.H., Hille J., Dijkwel P.P. (2005). Ethylene-induced leaf senescence depends on age-related changes and OLD genes in *Arabidopsis*. J. Exp. Bot..

[18] Buchanan-Wollaston V., Page T., Harrison E., Breeze E., Lim P.O., Nam H.G., Lin J.-F., Wu S.-H., Swidzinski J., Ishizaki K. (2005). Comparative transcriptome analysis reveals significant differences in gene expression and signalling pathways between developmental and dark/starvation-induced senescence in *Arabidopsis*. Plant J..

[19] Kim H.J., Hong S.H., Kim Y.W., Lee I.H., Jun J.H., Phee B.K., Rupak T., Jeong H., Lee Y., Hong B.S. (2014). Gene regulatory cascade of senescence-associated NAC transcription factors activated by ETHYLENE-INSENSITIVE2-mediated leaf senescence signalling in *Arabidopsis*. J. Exp. Bot..

[20] Grbic V., Bleecker A.B. (1995). Ethylene regulates the timing of leaf senescence in *Arabidopsis*. Plant J..

[21] Finkelstein R. (2013). Abscisic acid synthesis and response. Arabidopsis Book.

[22] Mori I.C., Murata Y., Yang Y., Munemasa S., Wang Y.-F., Andreoli S., Tiriac H., Alonso J.M., Harper J.F., Ecker J.R. (2006). CDPKs CPK6 and CPK3 function in ABA regulation of guard cell S-type anion- and Ca(2+)-permeable channels and stomatal closure. PLoS Biol..

[23] Munemasa S., Muroyama D., Nagahashi H., Nakamura Y., Mori I.C., Murata Y. (2013). Regulation of reactive oxygen species-mediated abscisic acid signaling in guard cells and drought tolerance by glutathione. Front. Plant Sci..

[24] Zhang K., Xia X., Zhang Y., Gan S.S. (2012). An ABA-regulated and Golgi-localized protein phosphatase controls water loss during leaf senescence in *Arabidopsis*. Plant J..

[25] Zhao Y., Chan Z., Gao J., Xing L., Cao M., Yu C., Hu Y., You J., Shi H., Zhu Y. (2016). ABA receptor PYL9 promotes drought resistance and leaf senescence. Proc. Natl. Acad. Sci. USA.

[26] Zhao Y., Gao J., Im Kim J., Chen K., Bressan R.A., Zhu J.-K. (2017). Control of plant water use by ABA induction of senescence and dormancy: An overlooked lesson from evolution. Plant Cell Physiol..

[27] Zhang K., Gan S.-S. (2012). An abscisic acid-AtNAP transcription factor-SAG113 protein phosphatase 2C regulatory chain for controlling dehydration in senescing *Arabidopsis* leaves. Plant Physiol..

[28] Planas-Riverola A., Gupta A., Betegón-Putze I., Bosch N., Ibañes M., Caño-Delgado A.I. (2019). Brassinosteroid signaling in plant development and adaptation to stress. Development.

[29] Hasan S.A., Hayat S., Ahmad A. (2011). Brassinosteroids protect photosynthetic machinery against the cadmium induced oxidative stress in two tomato cultivars. Chemosphere.

[30] Saglam-Cag S. (2007). The effect of epibrassinolide on senescence in wheat leaves. Biotechnol. Biotechnol. Equip..

[31] Li Z., Pan X., Guo X., Fan K., Lin W. (2019). Physiological and transcriptome analyses of early leaf senescence for ospls1 Mutant rice (*Oryza sativa* L.) during the grain-filling stage. Int. J. Mol. Sci..

[32] Fedina E., Yarin A., Mukhitova F., Blufard A., Chechetkin I. (2017). Brassinosteroid-induced changes of lipid composition in leaves of *Pisum sativum* L. during senescence. Steroids.

[33] Wasternack C., Hause B. (2013). Jasmonates: Biosynthesis, perception, signal transduction and action in plant stress response, growth and development. An update to the 2007 review in annals of botany. Ann. Bot..

[34] Ueda J., Kato J. (1981). Promotive effect of methyl jasmonate on oat leaf senescence in the light. Z. Pflanzenphysiol..

[35] He Y., Tang W., Swain J.D., Green A.L., Jack T.P., Gan S. (2001). Networking senescence-regulating pathways by using *Arabidopsis* enhancer trap lines. Plant Physiol..

[36] He Y., Fukushige H., Hildebrand D.F., Gan S. (2002). Evidence supporting a role of jasmonic acid in *Arabidopsis* leaf senescence. Plant Physiol..

[37] Breeze E., Harrison E., McHattie S., Hughes L., Hickman R., Hill C., Kiddle S., Kim Y.S., Penfold C.A., Jenkins D. (2011). High-resolution temporal profiling of transcripts during *Arabidopsis* leaf senescence reveals a distinct chronology of processes and regulation. Plant Cell.

[38] Jung C., Lyou S.H., Yeu S., Kim M.A., Rhee S., Kim M., Lee J.S., Choi Y.D., Cheong J.-J. (2007). Microarray-based screening of jasmonate-responsive genes in *Arabidopsis thaliana*. Plant Cell Rep..

[39] Xiao S., Dai L., Liu F., Wang Z., Peng W., Xie D. (2004). COS1: An *Arabidopsis coronatine insensitive1* suppressor essential for regulation of jasmonate-mediated plant defense and senescence. Plant Cell.

[40] Tan X.-L., Fan Z.-Q., Shan W., Yin X.-R., Kuang J.-F., Lu W.-J., Chen J.-Y. (2018). Association of BrERF72 with methyl jasmonate-induced leaf senescence of Chinese flowering cabbage through activating JA biosynthesis-related genes. Hortic. Res..

[41] Vlot A.C., Dempsey D.M.A., Klessig D.F. (2009). Salicylic Acid, a Multifaceted Hormone to Combat Disease. Annu. Rev. Phytopathol..

[42] Besseau S., Li J., Palva E.T. (2012). WRKY54 and WRKY70 co-operate as negative regulators of leaf senescence in *Arabidopsis thaliana*. J. Exp. Bot..

[43] Guo P., Li Z., Huang P., Li B., Fang S., Chu J., Guo H. (2017). A tripartite amplification loop involving the transcription factor WRKY75, salicylic acid, and reactive oxygen species accelerates leaf senescence. Plant Cell.

[44] Gan S., Amasino R.M. (1995). Inhibition of leaf senescence by autoregulated production of cytokinin. Science.

[45] Guo Y., Gan S.S. (2014). Translational researches on leaf senescence for enhancing plant productivity and quality. J. Exp. Bot..

[46] Merewitz E.B., Gianfagna T., Huang B. (2011). Protein accumulation in leaves and roots associated with improved drought tolerance in creeping bentgrass expressing an ipt gene for cytokinin synthesis. J. Exp. Bot..

[47] Rivero R.M., Gimeno J., Van Deynze A., Walia H., Blumwald E. (2010). Enhanced cytokinin synthesis in tobacco plants expressing PSARK::IPT prevents the degradation of photosynthetic protein complexes during drought. Plant Cell Physiol..

[48] Hwang I., Chen H.-C., Sheen J. (2002). Two-component signal transduction pathways in *Arabidopsis*. Plant Physiol..

[49] Kim H.J., Ryu H., Hong S.H., Woo H.R., Lim P.O., Lee I.C., Sheen J., Nam H.G., Hwang I. (2006). Cytokinin-mediated control of leaf longevity by AHK3 through phosphorylation of ARR2 in *Arabidopsis*. Proc. Natl. Acad. Sci. USA.

[50] Brenner W.G., Romanov G.A., Kollmer I., Burkle L., Schmulling T. (2005). Immediate-early and delayed cytokinin response genes of *Arabidopsis thaliana* identified by genome-wide expression profiling reveal novel cytokinin-sensitive processes and suggest cytokinin action through transcriptional cascades. Plant J..

[51] Ali A., Gao X., Guo Y. (2018). Initiation, progression, and genetic manipulation of leaf senescence. Plant Senescence.

[52] Noh Y.S., Amasino R.M. (1999). Identification of a promoter region responsible for the senescence-specific expression of SAG12. Plant Mol. Biol..

[53] Kim J.I., Murphy A.S., Baek D., Lee S.W., Yun D.J., Bressan R.A., Narasimhan M.L. (2011). YUCCA6 over-expression demonstrates auxin function in delaying leaf senescence in *Arabidopsis thaliana*. J. Exp. Bot..

[54] Kant S., Bi Y.-M., Zhu T., Rothstein S.J. (2009). SAUR39, a small auxin-up RNA gene, acts as a negative regulator of auxin synthesis and transport in rice. Plant Physiol..

[55] Ellis C.M., Nagpal P., Young J.C., Hagen G., Guilfoyle T.J., Reed J.W. (2005). AUXIN RESPONSE FACTOR1 and AUXIN RESPONSE FACTOR2 regulate senescence and floral organ abscission in *Arabidopsis thaliana*. Development.

[56] Lim P.O., Lee I.C., Kim J., Kim H.J., Ryu J.S., Woo H.R., Nam H.G. (2010). Auxin response factor 2 (ARF2) plays a major role in regulating auxin-mediated leaf longevity. J. Exp. Bot..

[57] Quirino B.F., Normanly J., Amasino R.M. (1999). Diverse range of gene activity during *Arabidopsis thaliana* leaf senescence includes pathogen-independent induction of defense-related genes. Plant Mol. Biol..

[58] Aharoni N., Anderson J.D., Lieberman M. (1979). Production and action of ethylene in senescing leaf discs: Effect of indoleacetic Acid, kinetin, silver ion, and carbon dioxide. Plant Physiol..

[59] Rodrigues C., Vandenberghe L.P.D., de Oliveira J., Soccol C.R. (2012). New perspectives of gibberellic acid production: A review. Crit. Rev. Biotechnol..

[60] Sun T.-P., Gubler F. (2004). Molecular mechanism of gibberellin signaling in plants. Annu. Rev. Plant Biol..

[61] Fletcher R.A., Osborne D.J. (1965). Regulation of protein and nucleic acid synthesis by gibberellin during leaf senescence. Nature.

[62] Li J.R., Yu K., Wei J.R., Ma Q., Wang B.Q., Yu D. (2010). Gibberellin retards chlorophyll degradation during senescence of Paris polyphylla. Biol. Plant..

[63] Yu K., Wang Y., Wei J., Ma Q., Yu D., Li J. (2009). Improving rhizome yield and quality of *Paris polyphylla* through gibberellic acid-induced retardation of senescence of aerial parts. Plant Signal. Behav..

[64] Guo Y. (2013). Towards systems biological understanding of leaf senescence. Plant Mol. Biol..

[65] Sekhon R.S., Saski C., Kumar R., Flinn B.S., Luo F., Beissinger T.M., Ackerman A.J., Breitzman M.W., Bridges W.C., de Leon N. (2019). Integrated genome-scale analysis identifies novel genes and networks underlying senescence in maize. Plant Cell.

[66] Guo Y., Gan S. (2005). Leaf senescence: Signals, execution, and regulation. Curr. Top. Dev. Biol..

[67] Li W., Guo Y.F. (2014). Transcriptome, transcription factors and transcriptional regulation of leaf senescence. J. Bioinforma. Comp. Genomics.

[68] Guo Y., Cai Z., Gan S. (2004). Transcriptome of *Arabidopsis* leaf senescence. Plant Cell Environ..

[69] Woo H.R., Koo H.J., Kim J., Jeong H., Yang J.O., Lee I.H., Jun J.H., Choi S.H., Park S.J., Kang B. (2016). Programming of plant leaf senescence with temporal and inter-organellar coordination of transcriptome in *Arabidopsis*. Plant Physiol..

[70] Balazadeh S., Riaño-Pachón D.M., Mueller-Roeber B. (2008). Transcription factors regulating leaf senescence in *Arabidopsis thaliana*. Plant Biol..

[71] Li W., Li X., Chao J., Zhang Z., Wang W., Guo Y. (2018). NAC family transcription factors in tobacco and their potential role in regulating leaf senescence. Front. Plant Sci..

[72] Shah S.T., Pang C.Y., Hussain A., Fan S.L., Song M.Z., Zamir R., Yu S.X. (2014). Molecular cloning and functional analysis of NAC family genes associated with leaf senescence and stresses in *Gossypium hirsutum* L.. Plant Cell Tissue Organ Cult..

[73] Kim H.J., Nam H.G., Lim P.O. (2016). Regulatory network of NAC transcription factors in leaf senescence. Curr. Opin. Plant Biol..

[74] Guo Y., Gan S. (2006). AtNAP, a NAC family transcription factor, has an important role in leaf senescence. Plant J..

[75] Balazadeh S., Kwasniewski M., Caldana C., Mehrnia M., Zanor M.I., Xue G.P., Mueller-Roeber B. (2011). ORS1, an H(2)O(2)-responsive NAC transcription factor, controls senescence in *Arabidopsis thaliana*. Mol. Plant.

[76] Garapati P., Xue G.-P., Munné-Bosch S., Balazadeh S. (2015). Transcription factor ATAF1 in *Arabidopsis* promotes senescence by direct regulation of key chloroplast maintenance and senescence transcriptional cascades. Plant Physiol..

[77] Wu A., Allu A.D., Garapati P., Siddiqui H., Dortay H., Zanor M.I., Asensi-Fabado M.A., Munne-Bosch S., Antonio C., Tohge T. (2012). JUNGBRUNNEN1, a reactive oxygen species-responsive NAC transcription factor, regulates longevity in *Arabidopsis*. Plant Cell.

[78] Yang S.D., Seo P.J., Yoon H.K., Park C.M. (2011). The *Arabidopsis* NAC transcription factor VNI2 integrates abscisic acid signals into leaf senescence via the COR/RD genes. Plant Cell.

[79] Zhang W.Y., Xu Y.C., Li W.L., Yang L., Yue X., Zhang X.S., Zhao X.Y. (2014). Transcriptional analyses of natural leaf senescence in maize. PLoS ONE.

[80] Kim H.J., Park J.-H., Kim J., Kim J.J., Hong S., Kim J., Kim J.H., Woo H.R., Hyeon C., Lim P.O. (2018). Time-evolving genetic networks reveal a NAC troika that negatively regulates leaf senescence in *Arabidopsis*. Proc. Natl. Acad. Sci. USA.

[81] Eulgem T., Rushton P.J., Robatzek S., Somssich I.E. (2000). The WRKY superfamily of plant transcription factors. Trends Plant Sci..

[82] Robatzek S., Somssich I.E. (2002). Targets of AtWRKY6 regulation during plant senescence and pathogen defense. Genes Dev..

[83] Robatzek S., Somssich I.E. (2001). A new member of the *Arabidopsis* WRKY transcription factor family, AtWRKY6, is associated with both senescence- and defence-related processes. Plant J..

[84] Miao Y., Laun T., Zimmermann P., Zentgraf U. (2004). Targets of the WRKY53 transcription factor and its role during leaf senescence in *Arabidopsis*. Plant Mol. Biol..

[85] Woo H.R., Kim H.J., Nam H.G., Lim P.O. (2013). Plant leaf senescence and death—Regulation by multiple layers of control and implications for aging in general. J. Cell Sci..

[86] Zhou X., Jiang Y., Yu D. (2011). WRKY22 transcription factor mediates dark-induced leaf senescence in *Arabidopsis*. Mol. Cells.

[87] Sharabi-Schwager M., Samach A., Porat R. (2010). Overexpression of the CBF2 transcriptional activator in *Arabidopsis* counteracts hormone activation of leaf senescence. Plant Signal. Behav..

[88] Woo H.R., Kim J.H., Kim J., Kim J., Lee U., Song I.-J., Kim J.-H., Lee H.-Y., Nam H.G., Lim P.O. (2010). The RAV1 transcription factor positively regulates leaf senescence in *Arabidopsis*. J. Exp. Bot..

[89] Hopkins M., Taylor C., Liu Z., Ma F., McNamara L., Wang T.-W., Thompson J.E. (2007). Regulation and execution of molecular disassembly and catabolism during senescence. New Phytol..

[90] Zhou C., Cai Z., Guo Y., Gan S. (2009). An *Arabidopsis* mitogen-activated protein kinase cascade, MKK9-MPK6, plays a role in leaf senescence. Plant Physiol..

[91] Gish L.A., Clark S.E. (2011). The RLK/Pelle family of kinases. Plant J..

[92] Shiu S.H., Bleecker A.B. (2001). Receptor-like kinases from *Arabidopsis* form a monophyletic gene family related to animal receptor kinases. Proc. Natl. Acad. Sci. USA.

[93] Shiu S.H., Bleecker A.B. (2001). Plant receptor-like kinase gene family: Diversity, function, and signaling. Sci. STKE.

[94] Ouelhadj A., Kaminski M., Mittag M., Humbeck K. (2007). Receptor-like protein kinase HvLysMR1 of barley (*Hordeum vulgare* L.) is induced during leaf senescence and heavy metal stress. J. Exp. Bot..

[95] Shiu S.-H., Karlowski W.M., Pan R., Tzeng Y.-H., Mayer K.F.X., Li W.-H. (2004). Comparative analysis of the receptor-like kinase family in *Arabidopsis* and rice. Plant Cell.

[96] Goff K.E., Ramonell K.M. (2007). The role and regulation of receptor-like kinases in plant defense. Gene Regul. Syst. Biol..

[97] Cock J.M., Vanoosthuyse V., Gaude T. (2002). Receptor kinase signalling in plants and animals: Distinct molecular systems with mechanistic similarities. Curr. Opin. Cell Biol..

[98] Wei Z., Wang J., Yang S., Song Y. (2015). Identification and expression analysis of the LRR-RLK gene family in tomato (*Solanum lycopersicum*) Heinz 1706. Genome.

[99] Zan Y., Ji Y., Zhang Y., Yang S., Song Y., Wang J. (2013). Genome-wide identification, characterization and expression analysis of populus leucine-rich repeat receptor-like protein kinase genes. BMC Genomics.

[100] Li X., Salman A., Guo C., Yu J., Cao S., Gao X., Li W., Li H., Guo Y. (2018). Identification and characterization of LRR-RLK family genes in potato reveal their involvement in peptide signaling of cell fate decisions and biotic/abiotic stress responses. Cells.

[101] Hajouj T., Michelis R., Gepstein S. (2000). Cloning and characterization of a receptor-like protein kinase gene associated with senescence. Plant Physiol..

[102] Delatorre C.A., Cohen Y., Liu L., Peleg Z., Blumwald E. (2012). The regulation of the SARK promoter activity by hormones and environmental signals. Plant Sci..

[103] Li X.P., Gan R., Li P.L., Ma Y.Y., Zhang L.W., Zhang R., Wang Y., Wang N.N. (2006). Identification and functional characterization of a leucine-rich repeat receptor-like kinase gene that is involved in regulation of soybean leaf senescence. Plant Mol. Biol..

[104] Xu F., Meng T., Li P., Yu Y., Cui Y., Wang Y., Gong Q., Wang N.N. (2011). A soybean dual-specificity kinase, GmSARK, and its *Arabidopsis* homolog, AtSARK, regulate leaf senescence through synergistic actions of auxin and ethylene. Plant Physiol..

[105] Li P., Yang H., Liu G., Ma W., Li C., Huo H., He J., Liu L. (2018). PpSARK Regulates Moss Senescence and Salt Tolerance through ABA Related Pathway. Int. J. Mol. Sci..

[106] Lee I.C., Hong S.W., Whang S.S., Lim P.O., Nam H.G., Koo J.C. (2011). Age-dependent action of an ABA-inducible receptor kinase, RPK1, as a positive regulator of senescence in *Arabidopsis* leaves. Plant Cell Physiol..

[107] Li X., Ahmad S., Ali A., Guo C., Li H., Yu J., Zhang Y., Gao X., Guo Y. (2019). Characterization of somatic embryogenesis receptor-like kinase 4 as a negative regulator of leaf senescence in *Arabidopsis*. Cells.

[108] Brandt B., Hothorn M. (2016). SERK co-receptor kinases. Curr. Biol..

[109] Hu C., Zhu Y., Cui Y., Cheng K., Liang W., Wei Z., Zhu M., Yin H., Zeng L., Xiao Y. (2018). A group of receptor kinases are essential for CLAVATA signalling to maintain stem cell homeostasis. Nat. Plants.

[110] Cui Y., Hu C., Zhu Y., Cheng K., Li X., Wei Z., Xue L., Lin F., Shi H., Yi J. (2018). CIK receptor Kinases determine cell fate specification during early anther development in *Arabidopsis*. Plant Cell.

[111] Zeng D.-D., Yang C.-C., Qin R., Alamin M., Yue E.-K., Jin X.-L., Shi C.-H. (2018). A guanine insert in OsBBS1 leads to early leaf senescence and salt stress sensitivity in rice (*Oryza sativa* L.). Plant Cell Rep..

[112] Zhang M., Su J., Zhang Y., Xu J., Zhang S. (2018). Conveying endogenous and exogenous signals: MAPK cascades in plant growth and defense. Curr. Opin. Plant Biol..

[113] Berberich T., Sano H., Kusano T. (1999). Involvement of a MAP kinase, ZmMPK5, in senescence and recovery from low-temperature stress in maize. Mol. Gen. Genet. MGG.

[114] Wang S.-H., Lim J.-H., Kim S.-S., Cho S.-H., Yoo S.-C., Koh H.-J., Sakuraba Y., Paek N.-C. (2015). Mutation of SPOTTED LEAF3 (SPL3) impairs abscisic acid-responsive signalling and delays leaf senescence in rice. J. Exp. Bot..

[115] Tang D., Innes R.W. (2002). Overexpression of a kinase-deficient form of the EDR1 gene enhances powdery mildew resistance and ethylene-induced senescence in *Arabidopsis*. Plant J..

[116] Frye C.A., Tang D., Innes R.W. (2001). Negative regulation of defense responses in plants by a conserved MAPKK kinase. Proc. Natl. Acad. Sci. USA.

[117] Li Y., Chang Y., Zhao C., Yang H., Ren D. (2016). Expression of the inactive ZmMEK1 induces salicylic acid accumulation and salicylic acid-dependent leaf senescence. J. Integr. Plant Biol..

[118] Zhang Y., Liu J., Chai J., Xing D. (2015). Mitogen-activated protein kinase 6 mediates nuclear translocation of ORE3 to promote ORE9 gene expression in methyl jasmonate-induced leaf senescence. J. Exp. Bot..

[119] Miao Y., Laun T.M., Smykowski A., Zentgraf U. (2007). *Arabidopsis* MEKK1 can take a short cut: It can directly interact with senescence-related WRKY53 transcription factor on the protein level and can bind to its promoter. Plant Mol. Biol..

[120] Yue H., Nie S., Xing D. (2012). Over-expression of *Arabidopsis* Bax inhibitor-1 delays methyl jasmonate-induced leaf senescence by suppressing the activation of MAP kinase 6. J. Exp. Bot..

[121] Ren Y., Li Y., Jiang Y., Wu B., Miao Y. (2017). Phosphorylation of WHIRLY1 by CIPK14 Shifts Its Localization and Dual Functions in *Arabidopsis*. Mol. Plant.

[122] Miao Y., Jiang J., Ren Y., Zhao Z. (2013). The single-stranded DNA-binding protein WHIRLY1 represses WRKY53 expression and delays leaf senescence in a developmental stage-dependent manner in *Arabidopsis*. Plant Physiol..

[123] Xiao D., Cui Y., Xu F., Xu X., Gao G., Wang Y., Guo Z., Wang D., Wang N.N. (2015). SENESCENCE-SUPPRESSED PROTEIN PHOSPHOTASE directly interacts with the cytoplasmic domain of SENESCENCE-ASSOCIATED RECEPTOR-LIKE KINASE and negatively regulates leaf senescence in *Arabidopsis*. Plant Physiol..

